# A British understanding of racialised gaze-cueing in the context of implicit racial bias, explicit racial identity and self-esteem

**DOI:** 10.1177/17470218251335304

**Published:** 2025-04-09

**Authors:** Makayla Z. Ward, Ayoub Bouguettaya, Wieske van Zoest

**Affiliations:** 1 School of Psychology, University of Birmingham, Birmingham, UK; 2 Cedars-Sinai Medical Center, Los Angeles, CA, USA

**Keywords:** Gaze-cueing effect (GCE), implicit racial bias, racial identity, self-esteem, racialised gaze-cue, attention

## Abstract

Gaze-cueing is subject to social influences; people tend to shift their attention in the same direction as others, but this relationship may be affected by the observer’s race and the observed’s race. Evidence suggests that Italian (Black and White) and American (Black and White) populations show preferential ingroup gaze-cueing for White participants, but no preferential variance for Black participants. This experimental study aimed to examine the robustness of this effect within a British population, with a secondary aim of understanding processes behind racial differences via the use of implicit racial bias, explicit racial identity and self-esteem measures. Results revealed that Black participants showed an ingroup bias in gaze-cueing, whereas no such bias was observed for White participants, contradicting previous findings. The hypothesised secondary processes did not significantly influence the biases in gaze-cueing between groups. These findings emphasise intergroup variability’s importance in gaining a better understanding of how racialised gaze-cueing manifests across different populations.

## Introduction

Shifts of spatial attention can be triggered by social cues (Birmingham et al., 2008). In social interactions, individuals are likely to orient gaze in the same direction as others (Shepherd, 2010). The influence of gaze direction on spatial attention has a very strong automatic component, occurring even when cues are unpredictable and without strategic incentive (Emery, 2000). However, evidence suggests that contextual settings and group membership can influence the extent to which gaze-cueing occurs (Cassidy et al., 2019; Deaner et al., 2007; Foulsham et al., 2010; Ohlsen et al., 2013; Pavani et al., 2019). For example, individuals are more likely to follow the gaze of individuals with high social status compared to low status or dominance (Dalmaso et al., 2012; Foulsham et al., 2010; Jones et al., 2010; Weisbuch et al., 2017), and more likely to follow the gaze of ingroup political members compared to outgroup political members (Luizza et al., 2011). Furthermore, asymmetries in performance can exist between groups depending on racial group membership (Dalmaso et al., 2015; Pavan et al., 2011). Research within Italian (Black and White) and American (Black and White) populations show preferential ingroup gaze-cueing for White participants, but no preferential variance for Black participants (Cassidy et al., 2019; Pavan et al., 2011). Therefore, the extent to which gaze-cueing can be observed within an individual appears to be nuanced based on extraneous factors.

Gaze-cueing is typically measured via a central cueing paradigm, where eye direction presented in the centre of the display acts as a cue. The gaze-cueing effect (GCE) refers to the observation that participants are quicker to respond to a target when an observed gaze direction from a face is congruent with the location of the target (Bayliss & Tipper, 2006). The GCE has been used to investigate how different racial groups respond to other racial faces (Pavan et al., 2011; Zhang et al., 2020), with Black and White participants. Pavan et al. (2011) manipulated the context of Black and White faces, with either random presentation of all faces, or racial segregation in separate blocks. It was reasoned that the blocked presentation would make race less salient, allowing participants to focus better on the cue. The results revealed a GCE for both Black and White faces in White participants during the blocked presentation, but when both Black and White faces were presented in a mixed order within the same block, White participants showed a GCE for White faces only. However, Black participants maintained a GCE for both racial groups in both contexts. This result has been corroborated in Italian (Dalmaso et al., 2015) and American populations (Weisbuch et al., 2017). According to Pavan et al. (2011), the results suggest that the White participants demonstrate enhanced ingroup perception, but only in the racially diverse presentation of mixed blocks, a bias that does not exist for the Black participants. Similarly to the White participants in Pavan et al.’s (2011) study, Zhang et al. (2023) indicate Chinese participants show a heightened sensitivity for White faces over Asian faces in mixed blocks, but not racially segregated blocks; however, this effect was suggested to be driven by the perception of hegemonic social status of White people, rather than a racial membership. Sidanius et al. (2000) discussed the perpetuation of social hierarchies based on perceived dominance often afforded to the group with power. Whilst in the Western context of Pavan’s study, it may be stipulated that the results are due to Black people being considered of lower social status compared to their White counterparts (Castelli et al., 2009), in the Eastern context of Zhang’s study, the Chinese participants appear to place their White counterparts as being of a higher social status (Dalmaso et al., 2020)

### Potential processes behind racial differences

Racial bias refers to a preferential evaluation of the self and others by race and can involve a positive attitude towards the ingroup or a negative attitude towards the outgroup (Tajfel & Turner, 1986). A well-used method in evaluating racial bias is the Implicit Association Test (IAT; Greenwald et al., 1998). The IAT measures reaction time to evaluative associations to understand implicit cognition, without the confound of socially desirable responses (Fazio & Olson, 2003). The IAT prompts individuals to respond quickly to positive or negative attributes that are paired with a concept, such as religion, age or race. Research on the race IAT using Black and White faces or racially stereotypical names, has shown that Black participants elicit a less polarised ingroup preference compared to White participants (Nosek et al., 2002). This work supports the idea that White participants hold more of a racial ingroup preference in gaze-cueing compared to Black participants (e.g. Dalmaso et al., 2015).

So far, few studies have studied the IAT in combination with gaze-cueing (e.g. Cañadas et al., 2007; Luizza et al., 2011) For example, the work of Cañadas et al. (2007) looked at the impact of reliability on the GCE, and subsequent ratings on the IAT. They investigated whether participants’ racial bias captured by the IAT would change based on a predictable gaze direction. The idea is that predictable gaze-cues, which more often validly cue the location of the target, are perceived as more trustworthy (Manssuer et al., 2015), and implicit associations would subsequently be more positively biased to predictable cues compared to unpredictable cues. Indeed, in their work, Cañadas and colleagues concluded that racial bias against Black faces was reduced after participants observed Black faces consistently cueing the location of the target, and White faces consistently looking away from the target. These results support previous studies stipulating implicit negative associations can be reduced by showing positive representations of stigmatised groups (Dasgupta & Greenwald, 2001). Overall, this suggests that associations observed within the IAT are malleable and can be influenced by perceptions of eye gaze (see also, Capozzi & Kingstone, 2024).

Implicit measures are often accompanied by explicit measures due to the consideration of the two as separate constructs (Bar-Anan & Vianello, 2018; Buttrick et al., 2020). Measuring explicit racial identity, particularly in underrepresented ethnic populations, helps to comprehend the intersectionality of race within an identity. Cross’ (1971) model of racial identity includes facets like ethnic–racial salience, and ethnocentricity, to encompass subdivisions of racial identity (Worrell et al., 2019). An explicit measure of racial identity can provide further insight into how a racialised GCE may manifest in different racial and ethnic groups. For example, an unstable racial identity may include the internalisation of negative stereotypes (Dupree et al., 2021). This perception may extend to contextual factors deemed influential in the GCE, such as social status; for example, if Black people are considered to have a lower social status (Castelli et al., 2009; Foulsham et al., 2010), or a lower level of trustworthiness (Strachan et al., 2017). Further, connectedness with group membership has been shown to increase the GCE in favour of the ingroup (Luizza et al., 2011). Conversely, maintaining self-hatred, for example, has been evidenced to distance people from establishing a healthy racial identity and favourable ingroup perceptions (Cokley, 2002).

Similarly, self-esteem (Rosenberg, 1979) is also a candidate process potentially important to gaze-cueing. Evidence suggests that participants with lower self-esteem show a more robust GCE compared to those with higher self-esteem (Wilkowski et al., 2009). These results showed that those with low self-esteem were more likely to engage in social monitoring by shifting their attention in line with others. It is possible that racial differences may occur based on differences in self-esteem, as Twenge and Crocker (2002) report that Black people demonstrated higher self-esteem on self-report measures, than other racial groups. A positive correlation between racial identity and self-esteem has also been observed in how Black women evaluate Black and White models (Makkar & Strube, 1995), with more positive evaluations being reported with high racial identity and self-esteem scores on explicit measures. Therefore, it might be expected that White people, thought to have lower self-esteem than their Black counterparts, would be more attentive to gaze-aversion, contradicting prior results found in Italian and North American populations (Dalmaso et al., 2015; Pavan et al., 2011; Weisbuch et al., 2017).

### Present study

The first aim of this study is to investigate whether an asymmetric bias in racialised gaze-cueing extends to the British population in Black and White British participants. It is hypothesised that, similar to the Italian (Dalmaso et al., 2015; Pavan et al., 2011) and U.S. (Weisbuch et al., 2017) populations, White participants will show ingroup preference, with quicker responses to the averted gaze of White faces compared to Black faces. However, we expect Black British participants to show no racial preference in the gaze-cueing task.

To learn more about the potential processes underlying racial biases in gaze-cueing, the second aim of our study is to explore the influence of implicit racial bias, explicit racial identity and self-esteem on the GCE. It is hypothesised that those with higher implicit racial bias towards their ingroup would be more likely to show an ingroup preference in the gaze-cueing task. In contrast, Black participants, who are more likely to report higher racial identity (Worrell et al., 2019) are expected more likely to show an ingroup preference. It is also hypothesised that those with low self-esteem, regardless of racial identity, are more likely to attend to averted gaze (Wilkowski et al., 2009), though this may further interact with racial group (Twenge & Crocker, 2002).

## Methods

### Participants

One hundred twenty-two adult participants were recruited through Prolific, a voluntary online recruitment site, with the selection criteria of being British, self-identifying as White or Black and over 18 years of age. These filters were applied through the screening protocols on Prolific, and so the study was only available to those who fit the criteria. Participants were awarded at a rate of £7/hr in accordance with payment requirements on Prolific.

### Power analysis

This sample was motivated by a power analysis based on a previously reported three-way interaction between cue-target spatial congruency, stimulus racial group membership and participant racial group membership with an effect size of $\eta_{\text{p}}^{2}$ = .058 (Pavan et al., 2011). To obtain a desired statistical power of 0.90 based on these effect sizes, with an alpha value of .05, a minimum sample size of 32 was required. Therefore, our final sample of 112 was appropriate for evaluating the effects of these measures.

### Materials

The experiments were run through OpenSesame (v3.3.5; Mathôt et al., 2012) online through JATOS (Lange et al., 2015), which also connected the questionnaires from Qualtrics. The experiments were completed in full screen on participants’ personal devices. Participants were informed that their reaction time and accuracy would be measured in the experiments and were encouraged to minimise distractions.

#### Gaze-cueing experiment

**Figure 1.**
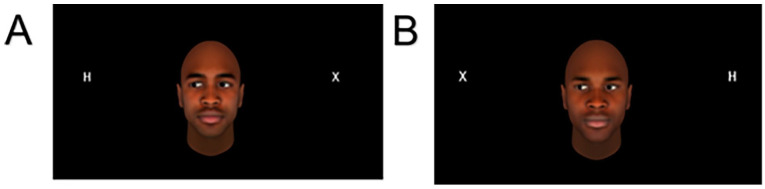
An example of the stimuli presented with the gaze-cueing experiment. *Note*. (A) depicts a valid cue (with eye gaze directed toward the target stimulus [‘H’]) and (B) depicts an invalid cue (with eye gaze directed away from the stimulus [‘H’]). In both cases, the correct response key is ‘m’. The letters were all capitalised in mono font.

The gaze-cueing experiment, replicated from Pavan et al. (2011), measures how quickly participants respond to a target stimulus, after observing a facial cue with directed gaze. Participants were instructed to respond with the ‘z’ key for the ‘F’ stimulus and ‘m’ key for the ‘H’ stimulus on their keyboard. The location of the stimulus was either valid or invalid with the direction of the gaze that precedes it, as shown in Figure 1. An ‘X’ appeared in the opposing location to the ‘F’ or ‘H’ target, to make the display symmetrical and limit the impact of the unique onset of only the target on reaction time (Schubö & Müller, 2009).

**Figure 2.**
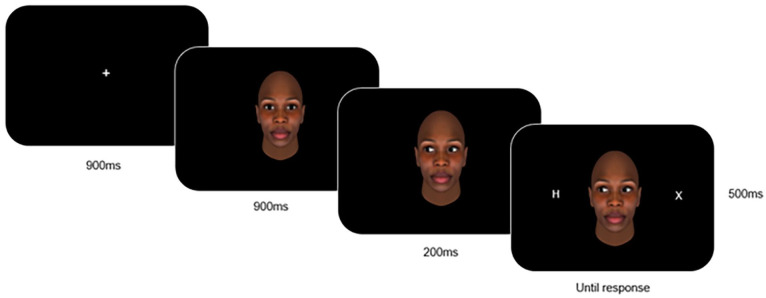
Gaze-cueing experiment procedure. *Note*. The text underneath the images denotes the duration of time the specific screen was presented to the participant. The central fixation cross was presented at the start of the trial for 900 ms, followed by the neutral gaze for 900 ms. The averted gaze-cue then appeared, with the target stimulus appearing 200 ms after. The stimulus remained onscreen until the participant responded, and the intertrial interval was 500 ms.

The stimuli set obtained from Pavan et al. (2011) includes 16 full-colour avatar faces, with four sets of Black female, Black male, White female and White male faces. Each set has a face with a neutral gaze with eye gaze directed forwards, and two averted gazes in either horizontal direction. These stimuli were presented as seen in Figure 2, in a random order with eight blocks of 32 trials, preceded by 16 practice trials. Participants were informed that the location of targets and direction of gaze was random, to avoid strategic processing (Wilkowski et al., 2009), and were instructed to focus on the centre of the screen where the fixation point resides (Pavan et al., 2011).

A trial started with the presentation of a fixation point for 900 ms, followed by a neutral face looking straight ahead for 900 ms. The gaze-cue was subsequently presented for 200 ms. A short inter-stimulus interval between the cue and the target presentation was used to measure the more reflexive responses to the cue instead of voluntary biases that can occur after longer intervals (Müller & Rabbitt, 1989). The stimulus remained onscreen until the participant responded or for a total duration of 2,000 ms. The intertrial interval was 500 ms.

#### Implicit bias

**Figure 3.**
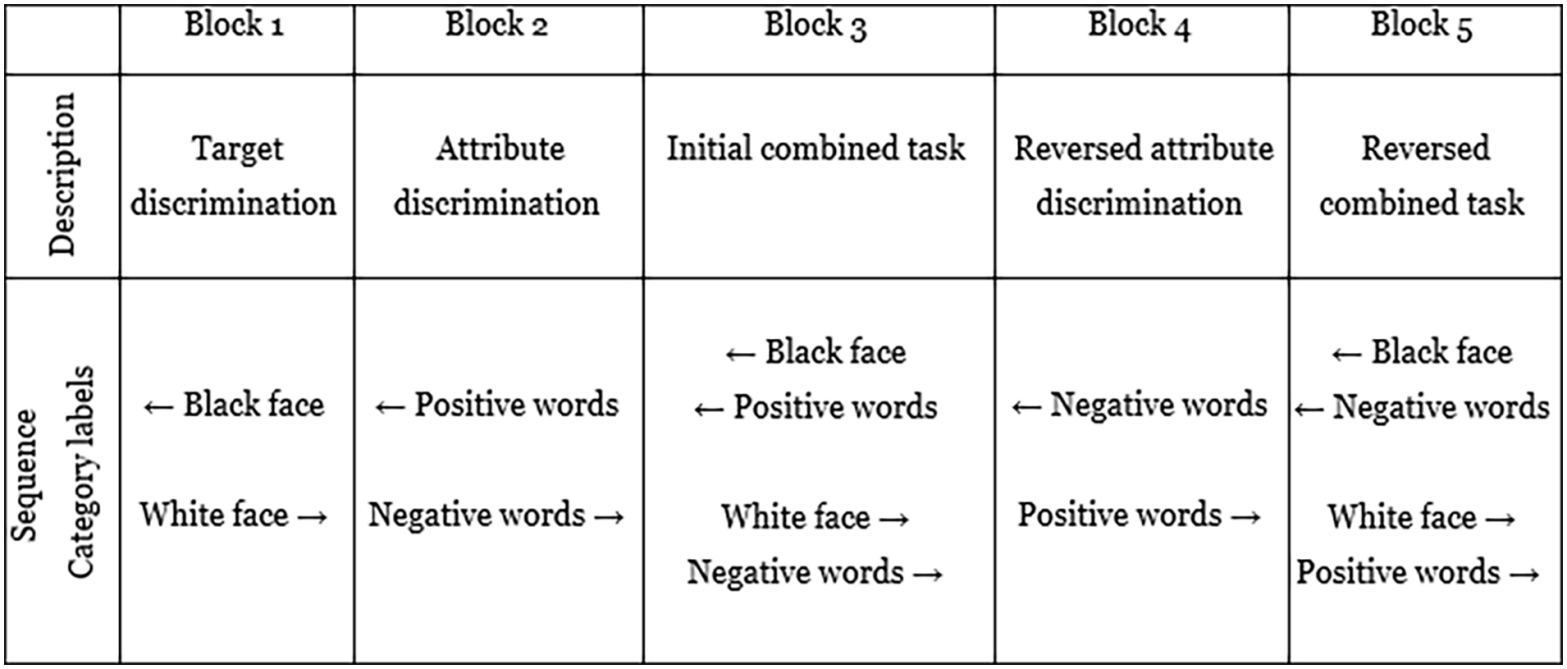
Implicit association test procedure. *Note*. ← and → refer to the direction the participant was instructed to respond, corresponding to the ‘E’ and ‘I’ keys on the keyboard, respectively. The sequence was counterbalanced so that half the participants were instructed to respond to the racial faces with inverse directions. Similarly, to the gaze-cueing experiment, there was a fixation cross that appeared for 500 ms before the stimuli were presented, the stimulus remained until the participant responded, and the intertrial interval was 500 ms. Blocks 1, 2 and 4 had 24 trials each, whilst the critical blocks which measured the evaluative paired associations, Blocks 3 and 5, had 72 trials each.

The race IAT (Greenwald et al., 1998) measured implicit racial bias through paired associations of Black and White facial stimuli and positive or negative words. Participants viewed the facial and word stimuli as defined by the corresponding blocks in Figure 3.

Within this experiment, there were 12 cropped photos of faces, with three sets of Black female, Black male, White female and White male faces (stimuli obtained from Project Implicit; Greenwald et al., 1998). The attribute stimuli were positive (happy, luck, joy, love, success and beauty) and negative words (horror, pain, fear, terror, disaster, illness). The category labels remained present for the entirety of the block, in congruence with the ‘E’ and ‘I’ response key orientation used for stimulus evaluation.

Implicit racial bias in favour of White faces is indicated by a faster reaction time when pairing White faces and positive words, and Black faces and negative words, relative to their contrary pairings. Results from this task were calculated using methods outlined in Greenwald et al.’s (2003) paper, and the scripts used can be found at https://osf.io/n34zu/.

#### Explicit bias

Worrell et al. (2019) developed the Cross Ethnic-Racial Identity Scale – Adult (CERIS-A) to measure racial identity strength, and the 29 items have been adapted to reflect the British population. A seven-point Likert scale was presented (1 = *strongly disagree*, 7 = *strongly agree*) where participants could indicate the level of agreement. To produce a score of racial identity strength from the seven subscales, the scoring within assimilation, miseducation, and self-hatred scales were reversed. Scores could range from 28 to 196, and a higher score indicates stronger racial identity strength.

#### Self-esteem

The Rosenberg (1979) Self-Esteem Scale – Revised Positive Version (SES) has 10 positive items (α = .962) adapted by Greenberger et al. (2003). A seven-point Likert scale was presented (1 = *strongly disagree*, 7 = *strongly agree*) where participants could indicate the level of agreement. Scores could range from 10 to 70, and a higher score indicates higher self-esteem.

### Procedure

After providing informed consent, participants were directed to the gaze-cueing experiment, followed by the IAT, the CERIS-A and finally the SES. At the study’s conclusion, participants were provided a debrief which included self-help resources relating to their individual relationship with race and ethnicity, and self-esteem.

## Results

Data were analysed using MATLAB R2020b (The Mathworks Inc., 2020) and SPSS v27 (IBM, 2020) . The final sample consisted of 112 participants, 56 self-identifying Black participants (34 female, *M* = 28.57 [*SD* = 5.994]) and 56 self-identifying White participants (41 female, *M* = 30.39 [*SD* = 5.148]). Ten participants (six Black (3 male, 3 female, *M* = 34.5, *SD* = 4.416), 4 White (1 male, 3 female, *M* = 30.5, *SD* = 5.568) were excluded based on providing less than 80% of valid data in the gaze-cueing experiment (Strachan et al., 2017). Invalid data was determined through incorrect trials (5.24% of total trials; Pavan et al., 2011) and trials with reaction times quicker than 100 ms (0.05% of total trials; Pavani et al., 2019) or trials slower than 2,000 ms (0.29% of total trials; Strachan et al., 2017). Overall accuracy was 94.7%.

**Figure 4.**
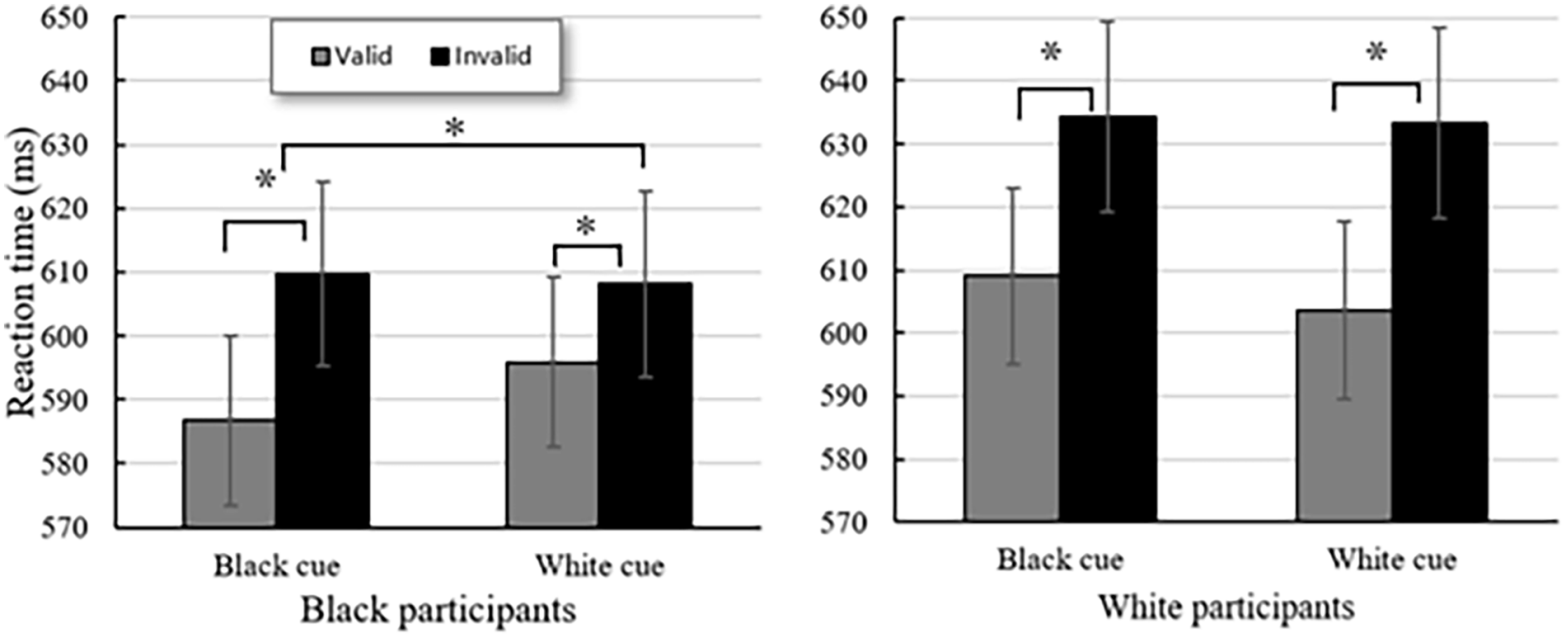
Results of the gaze-cueing experiment for Black and White participants. *Note*. *p < .005. The reaction times of the valid trials compared to the invalid trials, separated by the race of the cue, with standard error bars. Black participants showed a significant difference in reaction time for valid and invalid Black cues (*t*(55) = −4.233, *p* < .001) and for White cues (*t*(55) = −3.202, *p* = .001). White participants showed a significant difference in reaction time for both valid and invalid Black cues (*t*(55) = −4.377, *p* < .001) and White cues (*t*(55) = −4.815, *p* < .001). The two-way interaction between race of the cue and the race of the participant was reliable for Black participants (*F*(1,55) = 5.018, *p* = .029, $\eta_{\text{p}}^{2}$ = .084) but not for White participants (*F*(1,55) = .709, *p* = .403, $\eta_{\text{p}}^{2}$ = .013).

A 2 (Participant Race: Black, White) × 2 (Cue-Target Spatial Congruency: Valid, Invalid) × 2 (Race of the Cue: Black, White) mixed measures ANOVA was used to analyse reaction time in the gaze-cueing experiment. The main analysis showed a main effect of participant race, *F*(1, 110) = 1.02, *p* = .31. There was a significant main effect for validity (*F*(1,110) = 44.908, *p* < .001, $\eta_{\text{p}}^{2}$ = .290), showing that participants responded more quickly when the cue was valid compared to invalid. There was no significant main effect for race of cue (*F*(1,110) = .026, *p* = .873). However, there was a significant interaction between race of cue and participant race (*F*(1,110) = 4.800, *p* = .031, $\eta_{\text{p}}^{2}$ = .042) and also a significant three-way interaction between validity, race of cue and race of participant (*F*(1,110) = 4.555, *p* = .035, $\eta_{\text{p}}^{2}$ = .040). Secondary analyses showed a significant interaction between the validity and the race of the cue for Black participants (*F*(1,55) = 5.018, *p* = .029, $\eta_{\text{p}}^{2}$ = .084), whilst the same interaction for White participants was not significant (*F*(1,55) = .709, *p* = .403, $\eta_{\text{p}}^{2}$ = .013). Figure 4 depicts this interaction, showing that there is a difference based on the race of the participant, with Black participants showing a greater latency disparity on cue validity for Black cues compared to White cues. Additionally, comparing the validity effect for the White cues only with participant race as a between-group factor showed a reliable interaction, *F*(1,110) = 5.71, *p* = .019, $\eta_{\text{p}}^{2}$ = .049, while comparing group performances on the Black cue did not, *F*(1,110) < 1. This suggests the response to the White gaze-cue is moderated by race of observer, but not the Black gaze-cue.

**Figure 5.**
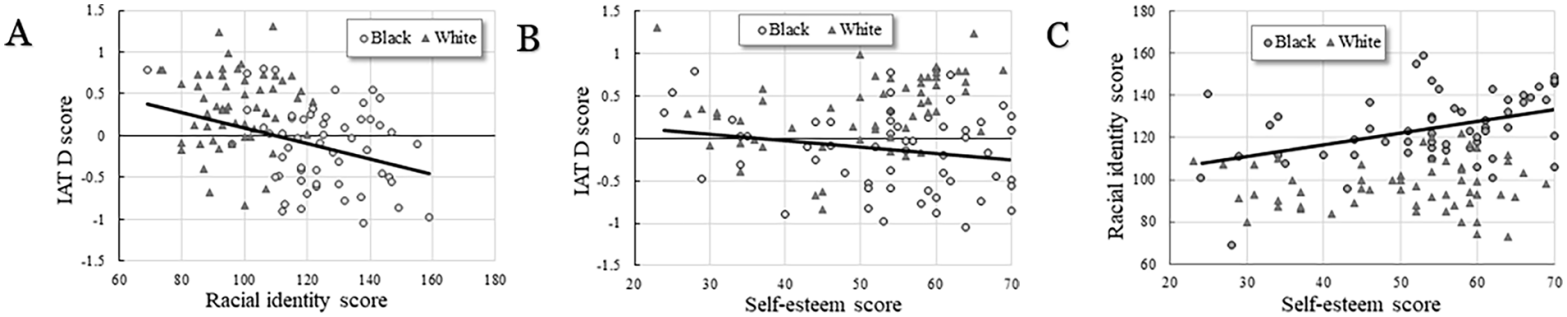
Graphs depicting the intercorrelations between the IAT, racial identity scores and self-esteem scores: (A) depicts the significant correlation between the IAT D score and racial identity score (R(110) = -.422, p <.001). (B). depicts the correlation between the IAT D score and self-esteem score (R(110) = -.022, p =.820, and (C) depicts the significant correlation between the racial identity and self-esteem score (R(110)=.294, p =.002). *Note*. Each correlation is separated by the self-identified race of the participants (circles representing Black participants and triangles represented White participants). IAT = Implicit Association Test.

Table 1 illustrates the group differences for the IAT, racial identity and self-esteem measures. The reaction times from the IAT are calculated from the combined tasks Blocks 3 and 5, where the difference in reaction times to the opposing associations is calculated. In the IAT, Black participants demonstrated quicker reaction times to Black and positive associations, and also White and negative associations, whilst White participants showed quicker reaction times to the contrary. Figure 5 depicts an examination of the correlations between these measures. (A) depicts the correlation between IAT D scores and racial identity strength scores (*r*(110) = −.422, *p* < .001), (B) depicts the correlation between IAT D scores and self-esteem scores(*r*(110) = −.022, *p* = .820) and (C) depicts the correlation between racial identity strength scores and self-esteem scores (*r*(110) = .294, *p* = .002).

**Table 1.** Descriptive statistics and analysis for the IAT, racial identity and self-esteem measures.

Measure	Black participants	White participants	T-test
IAT reaction time (ms)			
White + Positive and Black + Negative association	704.47 *(123.6)*	666.68 *(98.89)*	
Black + Positive and White + Negative association	672.88 (*91.36)*	736.74 *(114.3)*	
Reaction time difference	−31.59	70.06	
IAT D scores	−0.1324 *(0.4856)*	0.3356 *(0.4442)*	*t*(110) = −5.283, *p* < .001
Racial identity (CERIS-A) scores	124.45 *(16.35)*	97.23 *(11.44)*	*t*(98.431) = 10.205, *p* < .001
Self-esteem (SES) scores	53.86 *(12.32)*	50.30 *(11.68)*	*t*(110) = 1.567, *p* = .060

*Note*. The values within this table represent the mean and standard deviation (in italic font within the brackets) and *t*-tests were conducted to investigate group differences. For the racial identity score, Levene’s test for equal variances was not assumed, and the degrees of freedom were adjusted accordingly. IAT = Implicit Association Test; CERIS-A = Cross Ethnic-Racial Identity Scale – Adult; SES = Self-Esteem Scale.

A hierarchical multiple regression was conducted to understand whether the IAT, racial identity and self-esteem could predict racialised gaze-cue, which was calculated by subtracting the gaze-cue latencies with White cues from Black cues. Whilst Figure 5 indicates the IAT and racial identity, and racial identity and self-esteem are significantly correlated, the collinearity statistics, tolerance and variance inflation factor, were within accepted limits (Field, 2005); therefore, multicollinearity was disregarded.

A four-step hierarchical multiple regression (Table 2) was conducted with racialised GCE as the dependent variable. The self-identified race of the participant was entered at Step 1, the IAT D scores entered at Step 2, racial identity scores at Step 3 and self-esteem scores at Step 4. The IAT was entered first due to it being an implicit measure that captures more automatic cognition compared to the explicit measures of racial identity and self-esteem (Greenwald et al., 1998).

**Table 2.** Summary of hierarchical regression analysis for variables predicting the difference between GCE with the Black cue and the GCE with the White cue (racialised GCE).

Variable	β	*t*	*sr* ^2^	*R*	*R* ^2^	∆*R*^2^	*F*
Step 1*				.199	.040	.040	4.555
Race of participant	.199	2.134*	0.040				
Step 2*				.237	.056	.016	3.232
Race of participant	.64	2.529*	0.055				
IAT D score	−.143	−1.369	0.016				
Step 3**				.240	.058	.002	2.206
Race of participant	.301	2.246*	0.044				
IAT D score	−.134	−1.267	0.014				
Racial identity	.059	.450	0.002				
Step 4**				.287	.082	.024	2.395
Race of participant	.289	2.165*	0.040				
IAT D score	−.153	−1.442	0.018				
Racial identity	−.006	−.041	0.000				
Self-esteem	.165	1.687	0.024				

*Note*. The race of the participant (*t*(107) = 2.165, *p* = .033 at step four) uniquely explained 4% of the variation in the regression model predicting racialised GCE. The inclusion of all other factors approached significance (*F*(4,107) = 2.395, *p* = .055 at step four). GCE = gaze-cueing effect; IAT = Implicit Association Test.

* *p* < .05, **p < .1

## Discussion

This study investigated race-contingent biases in gaze-cueing in Black and White participants in a British population and explored whether implicit racial bias, explicit racial identity and self-esteem influenced the overall pattern of results. There were three main findings. First, the results showed reliable gaze-cueing across all groups and conditions, as has been observed in many other studies (Pavan et al., 2011; Strachan et al., 2017; Weisbuch et al., 2017; Zhang et al., 2020). Second, an ingroup bias in gaze-cueing was found for Black participants, but not for White participants, who showed no differences in performance to Black and White gaze-cues. Third, whilst significant differences between groups were found in implicit racial bias and explicit racial identity strength measures, the hierarchical regression model indicated that these variables did not significantly influence the biases in gaze-cueing between groups.

The present gaze-cueing experiment showed an ingroup preference for Black participants, where the susceptibility to the gaze-cue was larger for the Black gaze-cue compared to the White gaze-cue. White participants responded equally well to the cue regardless of the race of the gaze-cue. Whilst these results show an asymmetric bias in racialised gaze-cueing, this contrasts with previous studies which indicate an ingroup bias for White participants, but not for Black participants. An ingroup bias for White participants in previous research has been found in Italian samples (Dalmaso et al., 2015; Pavan et al., 2011) and a U.S. sample (Weisbuch et al., 2017). Notably, an absence of preferential looking in White British participants has been observed previously; however, this was during an investigation using White and East Asian gaze-cues (Strachan et al., 2017), with East Asian people potentially being subject to different racialised perceptions compared to Black people (Goh & McCue, 2021; Phills et al., 2018; Sudbury & Wilberforce, 2006; Yu et al., 2023).

Previous research into gaze-cueing has suggested that social rank and dominance help explain group differences in gaze-cueing (Jones et al., 2010). Some have suggested that different races are valued differently (Dalmaso et al., 2015), as typically White people are automatically placed on a higher social ranking over other ethnic groups (Christian, 2018; Sidanius et al., 2000). This is supported by research in implicit social dominance orientation, which encompasses a preference for social hierarchies compared to equality (Holt & Sweitzer, 2020), and racial bias, where higher levels of social dominance orientation are thought to predict higher implicit anti-Black bias (West et al., 2021). Additionally, the perception of a higher social status has also been observed to produce a higher attendance to eye gaze (Dalmaso et al., 2012; Foulsham et al., 2010; Ohlsen et al., 2013). Zhang et al. (2023) indicates that in Chinese participants, a gaze-cueing preference is observed towards White faces over Asian faces. This contradicts a preference for ingroup faces, and the authors suggest perceived social status of White faces driving these results in this population (Zhang et al., 2021). However, the present results show no evidence that the White cue was more influential than the Black cue and are not in line with the idea that White cues were perceived as more socially dominant. If racial bias had been symmetric across groups, both racial groups should be similarly influenced by their group membership (Luizza et al., 2011), and racial outgroup faces being less trusted than ingroup faces (Stanley et al., 2011) regardless of the specific racial membership. Capozzi and Kingstone (2024) highlight the importance of understanding interpersonal factors in how they may impact attention in social settings, which supports the notion of considering factors such as group membership, race and social status in gaze-cueing tasks.

Previous work has suggested that observers may treat avatars differently to photographs of human faces (Balas & Pacella, 2017). This work has shown that trustworthiness was rated lower for the avatars compared to the human faces. Knowing that trustworthiness may affect the gaze-cue effect (Cañadas et al., 2007, Strachan et al., 2017; Süßenbach & Schönbrodt, 2014), this may have underestimated the gaze-cue effect in the present study. Nonetheless, the present results find a very reliable gaze-cue effect across most conditions, suggesting that the avatars were effective in their ability to orient attention. In addition, a recent meta-analytic review on the GCE showed that this effect was reliably consistent regardless of whether schematic, real or computer-generated faces were used (McKay et al., 2021). The use of avatars in the present study, which extracts information on only the face, leaving out other personal features, allows for standardised viewing of every face. The benefit of using standardised avatar controls for any additional perception of personal features, which may be a confound influencing the GCE.

Though the results of the present study contrast the results from Pavan et al. (2011) using similar methods, along with a similar set of avatar-created facial stimuli, the control of a laboratory setting was not obtained in this work. In contrast to Pavan et al. (2011), the data for the present study were collected online, implying there was less control over extraneous conditions that may have affected the performance. Our results are consistent with a recent study conducted in Italy that collected data online, showing an ingroup preference for White Italian observers existing only for those who score high on the Autism Quotient (AQ; Ricciardelli & Pintori, 2023). The study failed to find an ingroup bias for White observers scoring low or average on the AQ measure. Though we did not take a measure of AQ, these results are in line with the results of the White group in the present study. Although there are notable differences between in-person and online testing (Buhrmester et al., 2016; Naglieri et al., 2004; Zhou & Fishbach, 2016), many experimental concepts have been successfully replicated across settings, indicating the possibility of strong reliability and validity using online formats (Arechar et al., 2018; Palan & Schitter, 2018; Ricciardelli & Pintori, 2023; Torrentira, 2020).

Furthermore, although both racial groups indicated an ingroup preference on the IAT, Black participants indicated a stronger identification with their racial identity compared to their White counterparts. This suggests a more prevalent intrapersonal connection relating to their race, which could also inform the reliable correlation found between the IAT and racial identity. While it makes sense that the increased ingroup bias for Black participants on the IAT explains the increased susceptibility to the Black gaze-cue of this group, the regression model revealed that variability in the race IAT did not explain performance on the gaze-cue task. This limitation of the IAT to predict performance and behaviour is in line with recent discussion regarding the external validity of the IAT. Even though the IAT has been shown to have good internal consistency (Gawronski & De Houwer, 2014), there has been a debate regarding the degree to which the results of the IAT are able to translate to explicit bias and behaviour (Röhner & Lai, 2020; Schimmack, 2021). The construct validity of the measure has been questioned, especially concerning the race IAT where only a small portion of the variance seems to reflect racial preferences (Schimmack, 2021). Similarly, Machery (2022) has argued that much of the research regarding implicit bias is oversimplified, as predictive results at an individual level poorly generalise to a much larger population (Rae & Olson, 2018). Schimmack and Howard (2021) have also pointed out that conclusions regarding implicit racial bias have historically been produced from the perspective of White researchers. Whilst they agree the IAT appears accurate in its reflection of implicit attitudes for White participants, they stipulate a low validity of the IAT for Black participants. The addition of other measures may be beneficial in supporting the ideas of robust connections between implicit measures and relevant behaviour (Kurdi et al., 2019). For example, Bar-Anan and Nosek (2014) propose alternative implicit measures such as the Affective Misattribution Procedure (Payne et al., 2005) and the Evaluative Priming Task (Fazio, 2007) can build on the IAT by separately obtaining data regarding ingroup and outgroup attitude strength.

Whilst the present work found a significant difference in racial identity between groups, and a positive correlation between racial identity and self-esteem across all participants, low intergroup variability in self-esteem was observed. As there was no significant difference in self-esteem across Black and White participants, this study has been unable to indicate results from previous studies linking self-esteem and gaze-cueing. Wilkowski et al. (2009) found that participants with low self-esteem are more strongly influenced by gaze-cue compared to participants with high self-esteem. This effect is thought to be driven by the feeling of belonging, often seen within ingroups with strong connectedness (Hughes et al., 2015). Based on evidence that suggests that Black people tend to demonstrate higher self-esteem on self-report measures than other racial groups (Twenge & Crocker, 2002), and also more evident racial identity strength, an interaction was predicted between racial identity and gaze-cueing. However, self-esteem did not correlate with the IAT for either group, which suggests that ingroup preferences do not necessarily extend to self-affect when comparing implicit and explicit measures (Forscher et al., 2019; Lai et al., 2014). Subsequently, neither implicit racial bias, racial identity or self-esteem were observed to predict gaze-cueing behaviours in this population.

Although the present research is specific to British participants, one limitation to consider is the prevalence of racial diversity surrounding individual participants. Much of the literature, particularly pertaining Black participants, has been conducted within American populations (Greenwald et al., 1998; Weisbuch et al., 2017; Worrell et al., 2019), or elsewhere in Europe (Dalmaso et al., 2020; Pavan et al., 2011). In America, self-identifying Black people are reported to represent approximately 14% of the national population as a racial minority, with White people representing 60% (Pew Research Center, 2021). In comparison, in England and Wales, self-identifying Black and White people are reported to represent 3.3% and 86% respectively, of the national population (Office for National Statistics, 2018). This may be important as racial disparity within an environment potentially influences how others view ethnic groups and racial minorities (Apfelbaum et al., 2008). In addition, different stereotypes may exist in the United Kingdom compared to the United States. Therefore, participants’ familiarity with Black or White people could affect the extent to which observers are susceptible to racial biases in gaze-cueing. However, because Black people in the Italian population are more likely to be exposed to White faces (ISTAT, 2015), Pavan et al. (2011) used racially ambiguous faces to study the role of familiarity. The idea was to compare responses to White faces to green-shaded multiracial faces in a gaze-cueing task, with White participants being more familiar with White faces and expecting greater GCEs. Pavan and colleagues concluded that familiarity did not modulate the GCE significantly enough to explain their asymmetric bias. These findings suggest there may be more processes behind the GCE in regard to race. However, it is important to consider that the Black participants involved in this study conducted in Italy were African students, as opposed to being Black Italians who would potentially have more familiarity with the racial diversity in Italy (Pavan et al., 2011). The present research required participants to identify as British and either Black or White. Further demographic detail was not obtained but gaining more insight into the environment’s participants surrounded themselves in could have been beneficial to understanding the mechanisms behind any group differences.

Investigating this further by understanding the variation in inter and intra-racial contact may influence understanding within this present scheme of work. Yip et al. (2010) explored the relationship between racial identity and intra-racial contact for Black students, concluding that higher intra-racial contact in a racially diverse environment allowed for stable racial identity and holding a greater sense of belonging to that group. Therefore, the authors posit that more opportunities for positive experiences lead to more favourable ingroup perceptions. This may consequently mean that the results of the GCE may have been moderated by both intra-racial and inter-racial contact for some people dependent on their racial environment.

Moreover, whilst the prevalence of Black people in Britain overall is 3.3%, this varies greatly when evaluating different geographical regions, as major cities tend to have more racial diversity than rural areas (Office for National Statistics, 2018). The overall experience can depend on the social context and environment. Accordingly, Black communities in the United States clearly report more positive experiences when surrounded by other Black people from childhood to university (Schimmack & Howard, 2021). Furthermore, the Black Lives Matter Movement in 2020 (McKersie, 2021; Pilkington, 2021; Schimmack & Howard, 2021; West et al., 2021) strongly influenced campaigns focusing on awareness and exposure to Black communities and disparities in status established throughout history (Joseph-Salisbury et al., 2020). This consequently may influence the strategies utilised in inter-racial social interactions (Apfelbaum et al., 2008; Berry, 2016; Leslie et al., 2019; Myers, 2019). The quality and quantity of intergroup contact are critical to holding positive implicit attitudes (Schmid et al., 2014; Vezzali et al., 2022). Therefore, understanding participants’ frequency of exposure to their ingroup within their communities, and their perception of diversity may support discourse surrounding group disparities (Wallrich et al., 2021).

### Conclusion

Our research demonstrates that gaze-cueing, a fundamental part of social attention, is affected by race of both the observer and the race of the viewed face in a way that is different from previous research. Our findings suggest racial biases in attention are malleable and susceptible to context. Further research that evaluates the processes behind racialised gaze-monitoring is critical to unfold the complexities of inter- and intra-racial social interactions. From a Western perspective, Eberhardt (2020) notes the importance of these interactions in society, with influence in a variety of settings, particularly for underrepresented ethnic groups. Future research should also examine the impact of the perception of social status as moderated by race as a function of cultural context or environment, on racialised gaze-cueing.
